# Antibody Response Induced by BNT162b2 and mRNA-1273 Vaccines against the SARS-CoV-2 in a Cohort of Healthcare Workers

**DOI:** 10.3390/v14061235

**Published:** 2022-06-07

**Authors:** Juan F. Delgado, Antoni Berenguer-Llergo, Germà Julià, Gema Navarro, Mateu Espasa, Sara Rodríguez, Noemí Sánchez, Eva Van Den Eynde, Marta Navarro, Joan Calvet, Jordi Gratacós, Rosa M. Serrano, Pilar Peña, María J. Amengual

**Affiliations:** 1 Immunology Section, Laboratory Service, Parc Taulí Hospital Universitari, Institut d’Investigació i Innovació Parc Taulí (I3PT), Universitat Autònoma de Barcelona, Departament de Medicina, 08208 Sabadell, Spain; gjulia@tauli.cat (G.J.); srodriguezna@tauli.cat (S.R.); nsanchezl@tauli.cat (N.S.); mjamengual@tauli.cat (M.J.A.); 2 Inflammatory Joint Diseases, Bone Metabolism, and Systemic Autoimmune Diseases Research Group, Parc Taulí Hospital Universitari, Institut d’Investigació i Innovació Parc Taulí (I3PT), Universitat Autònoma de Barcelona, 08208 Sabadell, Spain; aberenguerl@tauli.cat; 3 Epidemiology Service, Parc Taulí Hospital Universitari, Institut d’Investigació i Innovació Parc Taulí (I3PT), Universitat Autònoma de Barcelona, 08208 Sabadell, Spain; gnavarro@tauli.cat; 4 Microbiology Section, Laboratory Service, Parc Taulí Hospital Universitari, Institut d’Investigació i Innovació Parc Taulí (I3PT), Universitat Autònoma de Barcelona, 08208 Sabadell, Spain; mespasa@tauli.cat; 5 Infection Disease Department, Parc Taulí Hospital Universitari, Institut d’Investigació i Innovació Parc Taulí (I3PT), Universitat Autònoma de Barcelona, 08208 Sabadell, Spain; evandeneynde@tauli.cat (E.V.D.E.); mnavarro@tauli.cat (M.N.); 6 Rheumatology Service, Parc Taulí Hospital Universitari, Institut d’Investigació i Innovació Parc Taulí (I3PT), Universitat Autònoma de Barcelona, Departament de Medicina, 08208 Sabadell, Spain; jcalvet@tauli.cat (J.C.); jgratacos@tauli.cat (J.G.); 7 Occupational Health Department, Parc Taulí Hospital Universitari, Institut d’Investigació i Innovació Parc Taulí (I3PT), Universitat Autònoma de Barcelona, 08208 Sabadell, Spain; rserrano@tauli.cat (R.M.S.); ppena@tauli.cat (P.P.)

**Keywords:** antibodies, SARS-CoV-2, vaccine, mRNA-1273, BNT162b2

## Abstract

The aim of this study was to characterize the antibody response induced by SARS-CoV-2 mRNA vaccines in a cohort of healthcare workers. A total of 2247 serum samples were analyzed using the Elecsys^®^ Anti-SARS-CoV-2 S-test (Roche Diagnostics International Ltd., Rotkreuz, Switzerland). Sex, age, body mass index (BMI), arterial hypertension, smoking and time between infection and/or vaccination and serology were considered the confounding factors. Regarding the medians, subjects previously infected with SARS-CoV-2 who preserved their response to the nucleocapsid (N) protein showed higher humoral immunogenicity (BNT162b2: 6456.0 U/mL median; mRNA-1273: 2505.0 U/mL) compared with non-infected (BNT162b2: 867.0 U/mL; mRNA-1273: 2300.5 U/mL) and infected subjects with a lost response to N protein (BNT162b2: 2992.0 U/mL). After controlling for the confounders, a higher response was still observed for mRNA-1273 compared with BNT162b2 in uninfected individuals (FC = 2.35, *p* < 0.0001) but not in previously infected subjects (1.11 FC, *p* = 0.1862). The lowest levels of antibodies were detected in previously infected non-vaccinated individuals (39.4 U/mL). Clinical variables previously linked to poor prognoses regarding SARS-CoV-2 infection, such as age, BMI and arterial hypertension, were positively associated with increasing levels of anti-S protein antibody exclusively in infected subjects. The mRNA-1273 vaccine generated a higher antibody response to the S protein than BNT162b2 in non-infected subjects only.

## 1. Introduction

In December 2019, an outbreak of an unknown cause of pneumonia, later named coronavirus disease 2019 (COVID-19), started in Wuhan, China. The disease was attributed to a novel coronavirus called severe acute respiratory syndrome coronavirus 2 (SARS-CoV-2), which rapidly spread worldwide, impacted daily human activities, strained the healthcare system and brought high mortality around the world [1]. The clinical manifestations of COVID-19 patients range from mild non-specific symptoms to severe pneumonia with organ function damage. However, a substantial proportion of COVID-19 cases are reported as asymptomatic [2,3]. The most common symptoms of COVID-19 are fever, cough, fatigue, dyspnea, myalgia, sputum production and headache [2,3].

More than 300 vaccines are currently being investigated for their potential role in stemming the COVID-19 pandemic [4]. The first vaccines approved by the Food and Drug Administration and the European Medicines Agency were based on mRNA technology: BNT162b2 (Pfizer-BioNTech) and mRNA-1273 (Moderna) [5]. These mRNA vaccines showed an efficacy greater than 90% [6], achieving B and T cell memory and antibody responses after a vaccination schedule [7,8,9]. These schedules comprise two doses of BNT162b2 or mRNA-1273, administered within a 3- or 4-week interval, respectively. In non-infected subjects, an antibody response of intermediate-to-moderate intensity, depending on the studies, was reported after the first dose [10,11,12], while the second dose induces a boost of this response [10,11,12,13,14]. After infection, vaccination produces a greater response than that observed in non-infected individuals [10,14,15,16]. After the two-dose vaccination scheme, 95% of individuals generate neutralizing antibodies [10]. Moreover, these antibodies show a positive correlation with the total antibody titer induced by vaccination [10,17]. Recent studies analyzing the effect of the third dose on the neutralizing capacity of antibodies reveal that this booster effect is capable of significantly increasing neutralizing antibody titers for different SARS-CoV-2 variants [18,19].

Several studies analyzed the antibody response induced by mRNA vaccines regarding the patients’ infection status, with some of them reporting a higher response for mRNA-1273 compared with BNT162b2 [20,21,22,23,24,25]. However, none of these works evaluated this vaccine’s response in subjects previously infected by SARS-CoV-2 who had lost their response against the nucleocapsid protein (N protein) and, with it, a substantial part of the protection conferred by the infection. Other works reported the influence on the vaccine of different factors previously associated with the severity of the infection, namely, sex, age, obesity, arterial hypertension and smoking habit, providing results that show some inconsistencies across these studies [26,27,28,29,30,31,32,33,34,35]. Importantly, the time elapsed from vaccination and/or from infection to serology have a huge impact on the measurement of the antibody titer, but these data were not always controlled in the previous works.

In this study, we aimed to assess and compare the response against the SARS-CoV-2 spike protein (S protein) after vaccination with mRNA-1273 or BNT162b2 across a wide variety of subjects differing according to their SARS-CoV-2 infection and vaccination status. In doing so, we used a cohort of healthcare workers (HCWs) that included previously infected subjects with a conserved antibody response to N protein, individuals who lost their N protein response after SARS-CoV-2 infection and subjects with no previous contact with the virus. Factors that were previously reported to influence COVID-19 severity were evaluated and controlled in the comparisons, including the elapsed time between infection and/or vaccination and time of serology.

## 2. Materials and Methods

### 2.1. Study Design and Participants

This work was an observational, cross-sectional study performed on a cohort of HCWs at the Parc Taulí University Hospital (PTUH) in Sabadell (Spain), which aimed to study the antibody immune response after mRNA vaccination with BNT162b2 or mRNA-1273. The study protocol was approved by the Drug Research Ethics Committee of our Institution. A total of 2174 HCWs were included, consisting of subjects who were carrying out their work activity at our hospital at the time of recruitment (April to August 2020), and accepted and signed the informed consent to participate in the study. The subjects included physicians, nurses, nurse assistants, care assistants and social workers, as well as staff from other services, such as administration, TI, maintenance, dining service and research. The cohort was part of a seroprevalence study to estimate the proportion of the immunized population against the COVID-19 and their levels of antibody response. A total of 2247 serum samples were obtained. Out of them, 2098 were collected after vaccination with a 2-dose mRNA schedule (BNT162b2 or mRNA-1273), while 149 samples were obtained from subjects previously exposed to SARS-CoV-2 before their vaccination (Table 1).

For the analyses, the samples were stratified into 3 groups according to the SARS-CoV-2 infection status of the donor at the time of blood collection: (1) subjects who had overcome the COVID-19 and conserved the antibody response against N protein (infected/Nprot+, *n* = 627), (2) individuals that had suffered SARS-CoV-2 infection but had lost the antibody response against the N protein (infected/Nprot−, *n* = 27), and (3) subjects who had not been in contact with SARS-CoV-2 (non-infected, *n* = 1593). The vaccination status of the subjects (BNT162b2, mRNA-1273 or non-vaccinated) was also considered for comparisons between the groups. Two serum samples were obtained from 73 infected/Nprot+ subjects, which were extracted before and after their vaccination; the rest of the individuals contributed only one sample to the study, which was collected after the 2-dose vaccination schedule. Anti-N protein antibody titers were quantified at three time points for most of the subjects of the study, roughly corresponding to May 2020, December 2020 and June 2021. The serology history of anti-N protein antibodies was used to assess the subjects’ status regarding a previous infection of SARS-CoV-2 (infected vs. non-infected) and their preservation of the anti-N protein response in previously infected individuals at the time of anti-S antibody quantification to ensure a correct classification into the condition groups. The response to anti-S protein was measured at one single time point that corresponded to June 2021 for vaccinated subjects, and to May or December 2020 for non-vaccinated subjects.

### 2.2. Analysis of Antibody Response to SARS-CoV-2 S and N Proteins

The antibody response to SARS-CoV-2 S protein was measured using the Elecsys^®^ Anti-SARS-CoV-2 S test (quantitative), while the antibody response to SARS-CoV-2 N protein was measured using the Elecsys^®^ Anti-SARS-CoV-2 IgM/IgA/IgG test (Roche Diagnostics International Ltd., Rotkreuz, Switzerland, semi-quantitative) according to the manufacturer’s instructions. A lack of response to the N-protein was defined at anti-N protein antibody titers lower than one.

### 2.3. Clinical Variables

Sex, age, body mass index (BMI), arterial hypertension, smoking habit and time interval between infection and/or vaccination and serology were collected through a survey carried out for the study and from the database of the Occupational Health department of the PTUH.

### 2.4. Statistical Analysis

For descriptive purposes, the cohort was characterized using absolute and relative frequencies for categorical variables, while medians were used for numerical measurements. For the descriptive table, bootstrap intervals with a 95% confidence interval (95%CI, 1000 resamples) were estimated, and the statistical significances of group differences were assessed via permutations tests using the Kruskal–Wallis statistic (continuous) or the chi-squared test statistic for contingency tables (categorical variables). Resampling and permutation methods were chosen in this part of the analysis to correctly account for the variability due to donors providing more than one sample in our cohort. Quantitative differences in anti-S protein antibodies between groups were assessed using linear mixed-effects models, where donor variance was modeled as a random effect to account for intra-individual variability. To do so, the antibody titers were log2-transformed in order to fulfill the assumptions of the model. In addition to the univariate analysis, age, sex, BMI, arterial hypertension, smoking habit and time interval between infection and/or vaccination and serology were considered the confounding factors. Adjusted group means at the original scale (after undoing the log2 transformation) and fold changes (FCs) and their 95%CIs were retrieved from the model to express the magnitude of the effects. Statistical significance was assessed using Wald tests derived from the models. To control for the effect of time intervals in serological results, anti-S protein antibody values were corrected previously to the formal statistical analysis (two-step correction). To do so, for each condition group separately, we fitted a model to the anti-S antibody titers in which the interval times from infection and/or vaccination (where suitable), the vaccine type and the rest of the confounders were included as explanatory variables. The coefficient estimations associated with the time from infection and/or vaccination to serology were retrieved from the model and subtracted from the original anti-S antibody values. The resulting quantities were, by definition, an estimation of the anti-S antibody titers corrected by the effect induced by differences in the time from infection and/or vaccination. To conduct such a correction, the linear models were parametrized so that the anti-S antibody values computed in this way corresponded to value estimations at 240 days after infection and 120 days after vaccination. This was accomplished by centering the values of time from infection and time from vaccination to 240 and 120 days, respectively, before performing the model fitting. As a quality control, since this approach tends to overestimate the statistical significance in some situations, we repeated our statistical analyses after conducting the same a priori correction described above, but this time with no other explanatory variable in the models other than the time from infection and/or vaccination. Comparing results from the two procedures allowed us to check that our approach did not provide anti-conservative results with an impact on the conclusions of the work. Of note, this procedure also implicitly corrected for time elapsed from infection and vaccination, which is another parameter that was likely to influence the vaccine responses. As it was not possible to include the three parameters in the models at the same time, for clarity, we decided to use the ones that took the time of anti-S antibody measurement as reference (i.e., time from vaccination and from infection to serology). The results were represented graphically using a boxplot in which the group means and 95%CIs were also included after an adjustment using confounders. To evaluate the association of clinical variables with anti-S protein antibody levels within each subject group, we used an analogous model in which the interaction between the clinical variable and the antibody titer was added (one at a time). Statistical significance was set at the 5% threshold. All analyses were conducted using R [36].

## 3. Results

### 3.1. Antibody Response to SARS-CoV-2 S Protein

A total of 2247 serum samples were obtained from 2174 individuals. One hundred and forty-nine samples were drawn from non-vaccinated subjects previously infected by SARS-CoV-2. A total of 71% of samples were obtained from non-infected individuals (non-infected, *n* = 1593), while 28% corresponded to samples from infected individuals that conserved response against the N protein (infected/Nprot+, *n* = 627). The remaining 1% were samples drawn from infected individuals who had lost response to N protein (infected/Nprot−, *n* = 27). Of note, all the not-vaccinated samples in our data were provided by subjects previously infected with SARS-CoV-2 who conserved their response against the N protein, and all subjects in the infected/Nprot− group had been vaccinated with BNT162b2 (Table 1).

The median age of the cohort was 45.9 years old, although the infected/Nprot− group was substantially younger (37.8 years). Females were the majority in this study (80%) and represented a similar proportion of the subjects across groups. HCW groups were heterogeneous regarding the time interval between infection and serology, ranging from a median of 66 days in non-vaccinated infected/Nprot+ individuals to 406 days in the infected/Nprot− group. This heterogeneity was also observed for the time interval between vaccination and serology, ranging from a median of 85 days in infected/Nprot+ subjects vaccinated with mRNA-1273 to 125 days in the infected/Nprot− group vaccinated with BNT162b2. Importantly, the differences in the time interval were observed between individuals inoculated with vaccines from different manufacturers (BNT162b2 and mRNA-1273). Overall, the median time intervals between vaccine administration and serology were 119 days and 91 days for BNT162b2 and mRNA-1273, respectively (Table 1). Importantly, BNT162b2 was the only vaccine available at the beginning of the vaccination campaign in our hospital, which explains the differences in the times elapsed from inoculation and serology quantifications between the two mRNA vaccines. As expected, the times from vaccination and from infection were significantly associated with antibody titers, making necessary their statistical control for assessing group differences (Supplementary Figures S1 and S2).

**Figure 1:**
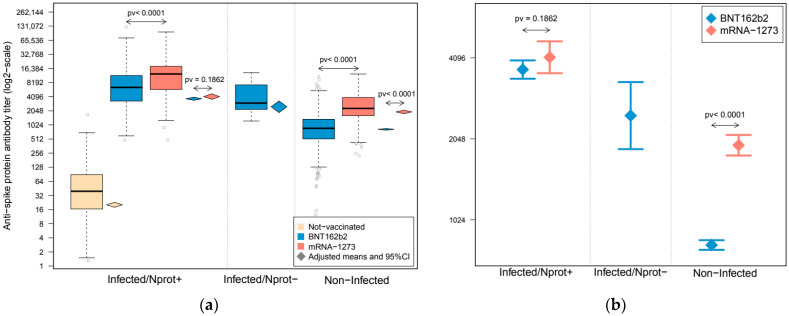
Antibody response against SARS-CoV-2 spike protein in a cohort of healthcare workers (HCWs). Subjects were grouped according to their infection and conservation status of antibody response against the nucleocapsid protein. Boxplots represent the distribution of anti-spike protein antibody quantification. (**a**) Upper and lower bounds of boxes indicate the 75th and 25th percentiles, respectively. Whiskers extend 1.5 times the interquartile range (IQR) from each extreme of the box. Diamond-shape symbols represent the adjusted group means of anti-spike protein antibody titer after statistical control for confounders, and their extension represents their 95% confidence intervals. (**b**) Adjusted means and 95% confidence intervals of the anti-spike protein antibody titer after statistical control for confounders in the vaccinated subject groups. Estimations were derived from a linear mixed-effect model in which the sample’s donor was modeled as a random effect to account for intra-individual variability, and were adjusted by sex, age, BMI, arterial hypertension, smoking habit and time interval from infection and/or vaccination to serology. Anti-spike protein antibody quantifications are expressed in a log2-scale.

The lowest levels for anti-S protein antibodies, a 39.4 U/mL median, were observed in non-vaccinated individuals (all included in the infected/Nprot+ group), which were remarkably lower than those observed in their vaccinated counterparts (6456.0 and 12,505.0 U/mL medians for BNT162b2 and mRNA-1273, respectively). Although at considerably lower levels, anti-S protein antibodies were also present in vaccinated subjects without previous contact with the SARS-CoV-2 (non-infected: 867 and 2300.5 U/mL medians for BNT162b2 and mRNA-1273, respectively) and for infected HCWs who had lost their response to the N protein (infected/Nprot−, all of them vaccinated with BNT162b2: 2992.0 U/mL) (Figure 1 and Table 1).

Comparative results of anti-S protein antibody levels between subject groups are shown in Figure 1 and Supplementary Table S1 (univariate associations) and Table 2 and Table S2 (adjusted by sex, age, BMI, arterial hypertension, smoking habit and time interval between infection and/or vaccination and serology). Non-infected subjects vaccinated with mRNA-1273 showed an antibody response that more than doubled that observed in non-infected individuals vaccinated with BNT162b2 (2.87 fold change (FC), *p* < 0.0001), even after adjusting for confounding factors (2.35 FC, *p* < 0.0001). Regarding previously infected individuals, a higher response was observed after mRNA-1273 vaccination (1.71 FC, *p* < 0.0001), which was similar to that observed after statistical control by sex, age, BMI, arterial hypertension and smoking habit time (FC = 1.69, *p*-value < 0.0001) or when additional control by time from infection to serology was added to the analyses (FC = 1.77, *p*-value < 0.0001). Nevertheless, these differences shrunk to 19% when adjusting for the time from vaccination to serology, in addition to sex, age, BMI, arterial hypertension and smoking habit (FC = 1.19, *p*-value = 0.0326), and decreased to a statistically insignificant 11% after controlling for all confounders, included time from infection and vaccination (FC = 1.11, *p*-value = 0.1862). Analyses stratified by age groups derived similar results (Supplementary Figure S3).

### 3.2. Association between Clinical Variables and Antibody Response to SARS-CoV-2 S Protein

Next, we explored the association of antibody titers with a set of clinical variables previously linked to poor prognosis in the course of SARS-CoV-2 infection (Table 3, see results detailed by vaccine type in Supplementary Table S3). A five-year increase in age in infected/Nprot+ subjects was found to be associated with a 7% increase in the amount of anti-S protein antibodies after vaccination (*p* = 0.0005). This association was also found in the non-vaccinated group, although it did not reach statistical significance (FC = 1.05, *p*-value = 0.0599) and, conversely, a decrease of roughly the same magnitude (6%) was observed in non-infected individuals (FC = −1.06, *p* < 0.0001). A 23% increase in anti-S protein antibody titers was also found for every five BMI points increase in non-vaccinated subjects (FC = 1.23, *p* < 0.0001), which was similar in infected/Nprot+ individuals (FC = 1.21, *p* < 0.0001). Nevertheless, this association was not found either in infected/Nprot− or in non-infected subjects. Arterial hypertension was also associated with an increase of antibody titers after vaccination, but only in infected/Nprot+ individuals (FC = 1.47, *p* = 0.0108). In general, subjects with a smoking habit showed less anti-S protein antibodies regardless their infection status (non-vaccinated: FC = −1.65, *p*-value = 0.0066; infected/Nprot+: FC = −1.61, *p* = 0.0001; infected/Nprot−: FC = −1.73, *p* = 0.0751; non-infected: FC = −1.32, *p* < 0.0001), although this decrease was substantially smaller and not statistically significant for subjects vaccinated with mRNA-1273 that had no previous contact with the SARS-CoV-2 (Supplementary Table S3). Finally, non-infected women vaccinated with BNT162b2 had a statistically significant increase of 18% in antibody titer compared with men in that group (*p* = 0.0021) which, surprisingly, was not observed in non-infected subjects vaccinated with mRNA-1273 (FC = 1.19, *p* = 0.1470, Supplementary Table S3).

## 4. Discussion

In this study, we described how levels of the antibody response to SARS-CoV-2 S protein were related to the number of exposures to the S protein in a cohort of HCWs. The lowest anti-S protein antibody titer corresponded to non-vaccinated individuals that had undergone COVID-19 infection (infected/Nprot+). Intermediate levels were found in vaccinated subjects without previous contact with SARS-CoV-2, which, in turn, were lower than those observed in infected and vaccinated individuals who had lost the antibody response to the N protein (infected/Nprot−). Finally, the highest levels of anti-S protein antibodies were displayed by vaccinated HCWs who conserved response to N protein (infected/Nprot+). It is well known that the secondary immune response is faster and more intense than the primary response and, therefore, our results should be interpreted under that context by taking into account both stimuli, vaccination and infection. Our work also allowed for evaluating the differences between secondary and tertiary immune responses to the SARS-CoV-2 by comparing anti-S protein antibody titers after vaccination from infected and non-infected subjects that provided results in agreement with previous studies [10,14,15,16].

Univariate analyses showed that HCWs vaccinated with mRNA-1273 had higher humoral immunogenicity to SARS-CoV-2 than those vaccinated with BNT162b2, in both previously infected and non-infected individuals. However, after adjusting for confounders, these differences persisted only in subjects without previous contact with the virus, while they were mainly explained by differences in the time interval between vaccination and serological determination in the infected/Nprot+ group. It must be highlighted that BNT162b2 was the only vaccine available at the beginning of the vaccination campaign in our hospital, which was the cause of these differences in time intervals between the two vaccines. That observation emphasized the need for controlling for the time from vaccination, apart from demographic characteristics, clinical parameters and time from infection, in order to make a correct assessment of the vaccines responses in serological studies.

The differences found in antibody titers of non-infected subjects vaccinated with BNT162b2 and mRNA-1273 might have been due to the higher mRNA concentration delivered in each dose of mRNA-1273 (100 µg vs. 30 µg) and/or to differences in the time interval between doses (3 or 4 weeks, respectively) [26]. Nevertheless, these differences were not observed in previously infected patients, where the second dose of vaccination represented the third encounter with the S protein of the SARS-CoV-2, as post-vaccination antibody levels were similar for both vaccines in this scenario. This result is in disagreement with a work published by Steensels et al. [20]. In their work, a significant 31% increase in antibody titer was reported in previously infected subjects vaccinated with mRNA-1273 compared with those inoculated with BNT162b2, while a similar difference to that reported in our study was observed in non-infected individuals. These discrepancies cannot be explained by differences in analytical techniques because the same reagents were used in both studies. However, they might have been due to differences in the strategy used for data analysis, as the time delay between vaccination and serology was not considered for the adjustment in their results, and the interaction between infection status and the vaccine type was not included in their multivariate model. To clarify this issue, further studies in larger cohorts are needed. In any case, both studies agreed that differences in the response between BNT162b2 and mRNA-1237 vaccines are lower when they were administered after COVID-19 infection. This observation suggested the existence of a plateau in the immune response that was reached in a third encounter with the S protein of SARS-CoV-2, and it would be expected that the response to a fourth exposure might be of a similar magnitude. This result supports the need for a third vaccination dose, following the current recommendations established by the governments and pharmaceutical companies, which include half a dose in the case of mRNA-1273. Further studies on subjects vaccinated with a third dose will be needed to corroborate this finding.

According to our results and after controlling for potential confounders, clinical variables previously linked to poor prognoses regarding SARS-CoV-2 infection, such as age, BMI and arterial hypertension, were positively associated with increasing levels of anti-S protein antibody titer after vaccination [27,28,29,30,31], but exclusively in infected/Nprot+ subjects. Although previous work reported that smoking harbors protection against SARS-CoV-2 infection, we and others have found lower antibody levels after vaccination associated with this habit [7,31,32]. Regarding sex, non-infected females vaccinated with BNT162b2 showed higher antibody titer in our study compared with males, which is in agreement with other published studies [33]. Intriguingly, this increase was not observed in individuals vaccinated with mRNA-1273, which is a fact that needs to be confirmed in further studies for a better evaluation of its clinical significance. The associations reported in our work between these clinical variables and post-vaccination antibody titers were reported in previous works [7,21,32,33,34,35], although we did not reproduce the association with BMI in non-infected subjects found by Pellini et al. [34].

The main weakness of this study was related to its cross-sectional design and the lack of baseline levels of anti-S antibodies before vaccination, which precluded an optimal control for the biological variability and prevents us to assess the vaccine response at the individual level. In the case of previously infected subjects with a conserved response to N protein (infected/Nprot+), this limitation was addressed in the methodology by accounting for the effects of potential confounders associated with the vaccine response (age, sex, BMI, arterial hypertension, smoking habit and time interval between infection or vaccination and serology) in the analyses. Theoretically, this strategy prevented us from introducing substantial biases due to baseline differences between the condition groups and allowed us to obtain estimations of the vaccine response at the group level. Regarding the rest of the subjects (infected/Nprot− and non-infected), the levels of anti-N protein antibodies were negative (<1) by definition and as confirmed by their serology history, and therefore, were expected to be their baseline levels of humoral immunogenicity against SARS-CoV-2. Therefore, anti-S protein antibody titers post-vaccination could be safely attributed to the response induced by the vaccine exclusively in these groups of subjects.

Another limitation of our study was its focus on HCWs, which, while heterogeneous from a socioeconomic point of view, overrepresented healthy individuals aged from 18 to 70 and females. Furthermore, the study was carried out in a single hospital (PTUH) and the lack of data from other clinical centers makes it difficult to extrapolate their results. From the start of the pandemic and similar to most Western European countries, the medical authorities set up a series of protocols and policies based on the epidemiology of the disease and aimed to minimize the spread of SARS-CoV-2 within the hospital, including respiratory protection with a surgical mask and the use of an FFP2/FFP3 when the risk of the situation required it, use of personal protective equipment for contact with positive patients, staff distancing, reduced capacity in meeting and work rooms, telecommuting of non-essential staff, ventilation of common areas for professionals and patients, and reduction and hospital closure regarding family visits. These measures have been maintained throughout the pandemic, except for family visits, which have been modified according to the incidence of the disease in the population at the given moment and the capacity of the areas shared with professionals. Despite these measures, the COVID-19 incidence among our healthcare workers was slightly higher: 65.1 in June 2021 (PTUH Occupational Health Risk department), compared with the 58.8 per 100,000 inhabitants in the hospital’s locality according to data published by the local authorities (Epidemiological Surveillance Service and Responses to Public Health Emergencies in Vallès Occidental and Vallès Oriental, 30 June 2021, report number 51). From our data, up to 23% of the subjects had overcome the infection by the middle of 2021, which corresponded to the time when most of the samples of our study were collected (93%). Finally, the study was carried out before data from a three-dose vaccination schedule was available for our series, which is currently recommended for mRNA vaccines according to previous studies and as suggested by our results (see above).

## 5. Conclusions

The level of immune response to SARS-CoV-2 increased with the number of times that the immune system was exposed to its S protein. The mRNA-1273 vaccine induced a greater intensity in the antibody response to the S protein than that observed for BNT162b2 in non-infected subjects that had received the two-dose vaccination schedule, while both vaccines induced a similar response in individuals that had already been exposed to the virus. Clinical variables previously linked to the poor prognosis of the COVID-19 infection were associated with the magnitude of the antibody response induced by vaccination in previously infected individuals.

## Figures and Tables

**Table 1:** Demographic and clinical characteristics of subjects in the study according to infection and vaccination status.

	All *N* = 2247 (2174 Subjects)	Infected/Nprot+ NotVacc *N* = 149 (6.6%)	Infected/Nprot+ BNT162b2 *N* = 361 (16.1%)	Infected/Nprot+ mRNA-1273 *N* = 117 (5.2%)	Infected/Nprot- BNT162b2 *N* = 27 (1.2%)	Non-Infected BNT162b2 *N* = 1303 (58.0%)	Non-Infected mRNA-1273 *N* = 290 (12.9%)	*p*-Value
**Sex, Female**	1741 (80.1%) [78.4%, 81.7%]	110 (73.8%) [67.1%, 80.5%]	287 (79.5%) [75.3%, 83.9%]	98 (83.8%) [76.1%, 89.7%]	21 (77.8%) [63.0%, 92.6%]	1044 (80.1%) [78.0%, 82.1%]	240 (82.8%) [78.3%, 87.2%]	0.3028
**Age**	45.9 [45.0, 46.6]	40.9 [38.2, 43.9]	44.3 [43.0, 46.4]	43.2 [41.4, 46.1]	37.8 [34.4, 41.8]	46.6 [45.7, 47.7]	47.8 [45.9, 50.2]	<0.0001
**Body Mass Index**	24.1 [23.9, 24.2]	23.6 [22.6, 24.3]	24.1 [23.4, 24.5]	25.0 [24.2, 25.7]	22.6 [21.5, 25.1]	24.1 [23.8, 24.4]	24.0 [23.4, 24.6]	0.0569
**Arterial hypertension**	175 (8.1%) [6.8%, 9.0%]	6 (4.3%) [1.4%, 7.9%]	27 (7.5%) [4.7%, 10.2%]	9 (7.7%) [3.4%, 12.8%]	1 (3.7%) [0.0%, 11.1%]	98 (7.5%) [6.1%, 8.9%]	36 (12.4%) [9.0%, 16.2%]	0.0405
**Smoking habit**	481 (22.2%) [20.2%, 23.7%]	18 (12.9%) [7.9%, 18.6%]	39 (10.8%) [7.8%, 14.1%]	16 (13.7%) [7.7%, 20.5%]	9 (33.3%) [14.8%, 51.9%]	317 (24.3%) [21.9%, 26.8%]	91 (31.4%) [26.2%, 37.2%]	<0.0001
**anti-N antibody titer**	0.1 [0.1, 0.1]	30.3 [22.4, 37.5]	18.1 [15.0, 22.8]	29.1 [19.9, 35.9]	0.5 [0.5, 0.7]	0.1 [0.1, 0.1]	0.1 [0.1, 0.1]	<0.0001
**Time from infection** **to serology (days)**	265.5 [256.5, 282.0]	66.0 [61.0, 74.0]	397.0 [378.0, 407.0]	370.0 [275.0, 395.0]	406.0 [398.0, 418.0]			<0.0001
**Time from vaccination** **to serology (days)**	114.0 [113.0, 115.0]		115.0 [113.0, 118.0]	85.0 [81.0, 91.0]	125.0 [110.0, 136.0]	120.0 [119.0, 121.0]	93.0 [91.0, 95.0]	<0.0001
**anti-S antibody titer**	1227.0 [1168.0, 1261.0]	39.4 [26.8, 45.4]	6456.0 [5909.3, 7292.0]	12505.0 [10137.0, 12505.0]	2992.0 [2297.0, 6323.0]	867.0 [830.0, 906.0]	2300.5 [2112.0, 2587.0]	<0.0001

Continuous variables are described by medians; categorical variables are summarized using absolute frequencies and percentages. Values between brackets are 95% confidence intervals (95%CIs) that were computed using bootstrapping (1.000 resamples). *p*-values were computed with permutation tests (10.000 permutations) using the statistic of a Kruskal–Wallis test (continuous variables) or a chi-squared test for contingency tables (categorical variables). N protein: nucleocapsid protein; S protein: spike protein; Infected/Nprot+: previously infected subjects with conserved response against N protein; Infected/Nprot−: previously infected subjects with a lost response against N protein; NotVacc: non-vaccinated subjects. *N*: number of samples.

**Table 2:** Pairwise comparisons of antibody response against spike protein between subject groups.

	Reference					
	Infected/Nprot+ NotVacc	Infected/Nprot+ BNT162b2	Infected/Nprot+ mRNA-1273	Infected/Nprot- BNT162b2	Non-Infected BNT162b2	Non-Infected mRNA-1273
**Infected/Nprot+** **NotVacc**		−183.01 [−209.66, −159.74] *p* < 0.0001	−203.27 [−242.23, −170.58] *p* < 0.0001	−123.44 [−168.44, −90.45] *p* < 0.0001	−40.75 [−46.35, −35.83] *p* < 0.0001	−95.80 [−111.43, −82.37] *p* < 0.0001
**Infected/Nprot+** **BNT162b2**	183.01 [159.74, 209.66] *p* < 0.0001		−1.11 [−1.30, 1.05] *p* = 0.1868	1.48 [1.10, 2.00] *p* = 0.0094	4.49 [4.11, 4.91] *p* < 0.0001	1.91 [1.70, 2.15] *p* < 0.0001
**Infected/Nprot+** **mRNA-1273**	203.27 [170.58, 242.23] *p* < 0.0001	1.11 [−1.05, 1.30] *p* = 0.1862		1.65 [1.20, 2.26] *p* = 0.0021	4.99 [4.33, 5.75] *p* < 0.0001	2.12 [1.80, 2.50] *p* < 0.0001
**Infected/Nprot-** **BNT162b2**	123.44 [90.45, 168.44] *p* < 0.0001	−1.48 [−2.00, −1.10] *p* = 0.0094	−1.65 [−2.26, −1.20] *p* = 0.0021		3.03 [2.27, 4.05] *p* < 0.0001	1.29 [−1.05, 1.74] *p* = 0.0977
**Non-Infected** **BNT162b2**	40.75 [35.83, 46.35] *p* < 0.0001	−4.49 [−4.91, −4.11] *p* < 0.0001	−4.99 [−5.75, −4.33] *p* < 0.0001	−3.03 [−4.05, −2.27] *p* < 0.0001		−2.35 [−2.59, −2.13] *p* < 0.0001
**Non-Infected** **mRNA-1273**	95.80 [82.37, 111.43] *p* < 0.0001	−1.91 [−2.15, −1.70] *p* < 0.0001	−2.12 [−2.50, −1.80] *p* < 0.0001	−1.29 [−1.74, 1.05] *p* = 0.0977	2.35 [2.13, 2.59] *p* < 0.0001	

The cells show the fold changes (FCs) and 95% confidence intervals (between brackets) observed between individual sets, which were estimated as the ratio between anti-spike protein antibodies levels in each group (rows) and that obtained from every other group defined in the study (columns, which are taken as a reference). Estimations are derived from a linear mixed-effect model in which the sample’s donor was modeled as a random effect to account for intra-individual variability, and were adjusted for sex, age, body mass index, arterial hypertension, smoking habit and time interval from infection and/or vaccination to serology. The anti-spike protein antibody titer was log2-transformed in order to fit the assumptions of the model. To facilitate the interpretation of the results, FCs lower than 1 were reversed (1/FC) and flagged with a minus sign (“−”). Infected/Nprot+: previously infected subjects with a conserved response against nucleocapsid protein; Infected/Nprot−: previously infected subjects with a lost response against nucleocapsid protein; NotVacc: non-vaccinated subjects; *p*: *p*-value.

**Table 3:** Fold changes (FCs), 95% confidence intervals (95%CI) and *p*-values for the association of antibody response against the spike protein with clinical variables previously linked to poor outcomes in SARS-CoV-2 infection.

	Sex Female		Age (Per 5 Years)		Body Mass Index (Per 5 Points)		Arterial Hypertension		Smoking Habit	
	FC [95%CI]	*p*-Value	FC [95%CI]	*p*-Value	FC [95%CI]	*p*-Value	FC [95%CI]	*p*-Value	FC [95%CI]	*p*-Value
**Infected/Nprot+** **NotVacc**	1.11 [−1.20, 1.46]	0.4774	1.05 [−1.00, 1.10]	0.0599	1.23 [1.14, 1.33]	<0.0001	1.12 [−1.21, 1.51]	0.4770	−1.65 [−2.36, −1.15]	0.0066
**Infected/Nprot+**	1.15 [−1.07, 1.42]	0.1809	1.07 1.03, 1.10]	0.0005	1.21 [1.11, 1.32]	<0.0001	1.47 [1.09, 1.98]	0.0108	−1.61 [−2.03, −1.27]	0.0001
**Infected/Nprot-**	1.87 [−1.07, 3.70]	0.0757	1.04 [−1.09, 1.17]	0.5878	1.02 [−1.49, 1.56]	0.91	−1.58 [−7.15, 2.88]	0.5549	−1.73 [−3.18, 1.06]	0.0751
**Non-Infected**	1.00 [−1.13, 1.14]	0.9467	−1.06 [−1.08, −1.04]	<0.0001	1.03 [−1.03, 1.09]	0.39	−1.02 [−1.19, 1.16]	0.8584	−1.32 [−1.46, −1.18]	<0.0001

For each variable, estimations were derived from a linear mixed-effects model in which the sample’s donor was modeled as a random effect to account for intra-individual variability, and were adjusted for the rest of the confounders (sex, age, body mass index, arterial hypertension, smoker habit and time intervals from infection and/or vaccination to serology). In each case, the model included the interaction with the variable to be evaluated in order to conduct comparisons within each of the subject groups. The anti-spike protein antibody titer was log2-transformed in order to fit the assumptions of the model. To facilitate the interpretation of the results, FCs lower than 1 were reversed (1/FC) and flagged with a minus sign (“−”). Infected/Nprot+: previously infected subjects with a conserved response against nucleocapsid protein; Infected/Nprot−: previously infected subjects with a lost response against nucleocapsid protein; NotVacc: non-vaccinated subjects; FC: fold change; 95%CI: 95% confidence interval.

## Data Availability

The data presented in this study are available on request from the corresponding author.

## Supplementary Materials

The following supporting information can be downloaded from https://www.mdpi.com/article/10.3390/v14061235/s1. Table S1: Univariate pairwise comparisons of antibody response against spike protein between subject groups. Table S2: Complete model for the assessment of antibody response against the spike protein between subject groups. Table S3: Fold changes (FCs), 95% confidence intervals (95%CIs) and *p*-values for the association of antibody response against the spike protein with clinical variables previously linked to poor outcomes regarding SARS-CoV-2 infection. Figure S1: Scatter plot showing the relationship of anti-spike antibody titers (log2 scale) with time from vaccination to serology (days) in SARS-CoV-2-vaccinated subjects. Figure S2: Scatter plot showing the relationship of anti-spike antibody titers (log2 scale) with time from infection to serology (days) in previously SARS-CoV-2-infected subjects. Figure S3: Antibody response against SARS-CoV-2 spike protein in cohort subjects aged from 18 to 34 (a), 35 to 54 (b) and 55 to 70 (c).
