# Supplementary material for: Antibody Response Induced by BNT162b2 and mRNA-1273 Vaccines against the SARS-CoV-2 in a Cohort of Healthcare Workers
Source: Viruses. 2022 Jun 7;14(6):1235. doi: 10.3390/v14061235 (PMC9229196; doi:10.3390/v14061235)
Supplement: Supplementary file 1 [file viruses-14-01235-s001.zip › viruses-1624687-supplementary.pdf]

Table S1. Univariate pairwise comparisons of antibody response against spike protein between subject groups.

	Reference					
	**Infected/Nprot+ NotVacc**	**Infected/Nprot+ BNT162b2**	**Infected/Nprot+ mRNA-1273**	**Infected/Nprot- BNT162b2**	**Non-Infected BNT162b2**	**Non-Infected mRNA-1273**
**Infected/Nprot+ NotVacc**		-164.93 [-190.20, -143.02] p < 0.0001	-281.50 [-339.33, -233.53] p < 0.0001	-101.01 [-142.04, -71.83] p < 0.0001	-21.75 [-24.89, -19.01] p < 0.0001	-62.37 [-73.14, -53.18] p < 0.0001
**Infected/Nprot+ BNT162b2**	164.93 [143.02, 190.20] p < 0.0001		-1.71 [-2.02, -1.44] p < 0.0001	1.63 [1.18, 2.27] p = 0.0034	7.58 [6.88, 8.35] p < 0.0001	2.64 [2.32, 3.01] p < 0.0001
**Infected/Nprot+ mRNA-1273**	281.50 [233.53, 339.33] p < 0.0001	1.71 [1.44, 2.02] p < 0.0001		2.79 [1.96, 3.95] p < 0.0001	12.94 [11.08, 15.12] p < 0.0001	4.51 [3.78, 5.39] p < 0.0001
**Infected/Nprot- BNT162b2**	101.01 [71.83, 142.04] p < 0.0001	-1.63 [-2.27, -1.18] p = 0.0034	-2.79 [-3.95, -1.96] p < 0.0001		4.64 [3.37, 6.39] p < 0.0001	1.62 [1.16, 2.25] p = 0.0043
**Non-Infected BNT162b2**	21.75 [19.01, 24.89] p < 0.0001	-7.58 [-8.35, -6.88] p < 0.0001	-12.94 [-15.12, -11.08] p < 0.0001	-4.64 [-6.39, -3.37] p < 0.0001		-2.87 [-3.19, -2.58] p < 0.0001
**Non-Infected mRNA-1273**	62.37 [53.18, 73.14] p < 0.0001	-2.64 [-3.01, -2.32] p < 0.0001	-4.51 [-5.39, -3.78] p < 0.0001	-1.62 [-2.25, -1.16] p = 0.0043	2.87 [2.58, 3.19] p < 0.0001	

The cells show the fold-changes (FC), 95% confidence intervals (between brackets) and p-values (p) observed between individual sets, which are estimated as the ratio between anti-spike protein antibodies titer obtained from serology in each group (rows) and that obtained from every other group defined in the study (columns, which are taken as reference). Estimations are derived from a linear mixed-effect model in which sample's donor was modeled as a random effect to account for intra-individual variability. Anti-spike protein antibody titer was log2-transformed in order ot fit the assumptions of the model. To facilitate the interpretation of the results, FCs lower than 1 have been reversed (1/FC) and flagged with a minus sign ("-"). **Infected/Nprot+:** previously infected subjects with conserved response against nucleocapsid protein; **Infected/Nprot-:** previously infected subjects with lost response against nucleocapsid protein; **NonVacc:** Non Vaccinated subjects; **p:** p-value.

Table S2. Complete model for assessment of antibody response against spike protein between subject groups.

	Groups	N(%) Median(IQR)	Adj.Means / Partial R2 [95%CI]	Coef. [95%CI]	Wald p-value	LRT p-value
Subject group	Infected Nprot+ NotVacc	140 (6.3%)	20.247 [17.929, 22.865]	Ref.		<0.0001
	Infected Nprot+ BNT162b2	361 (16.2%)	3705.386 [3426.452, 4007.027]	7.516 [7.320, 7.712]	<0.0001	
	Infected Nprot+ mRNA-1273	117 (5.2%)	4115.685 [3591.813, 4715.965]	7.667 [7.414, 7.920]	<0.0001	
	Infected Nprot- BNT162b2	27 (1.2%)	2499.218 [1876.349, 3328.854]	6.948 [6.499, 7.396]	<0.0001	
	Non-Infected BNT162b2	1301 (58.2%)	825.134 [791.700, 859.980]	5.349 [5.163, 5.535]	<0.0001	
	Non-Infected mRNA-1273	288 (12.9%)	1939.781 [1775.857, 2118.835]	6.582 [6.364, 6.800]	<0.0001	
Sex	Female	1791 (80.2%)	1036.072 [999.813, 1073.645]	Ref.		0.2440
	Male	443 (19.8%)	987.493 [918.771, 1061.355]	-0.069 [-0.186, 0.047]	0.2440	
Age (5 years)		9.17 3.530)	0.010 [0.003, 0.020]	-0.051 [-0.073, -0.030]	<0.0001	<0.0001
BMI (5 points)		4.82 (1.081)	0.005 [0.001, 0.013]	0.095 [0.040, 0.150]	0.0007	0.0007
Arterial hypertension	No	2057 (92.1%)	1022.197 [988.711, 1056.818]	Ref.		0.4294
	Yes	177 (7.9%)	1074.573 [954.597, 1209.629]	0.072 [-0.107, 0.251]	0.4294	
Smoker habit	Non smoker	1744 (78.1%)	1121.543 [1081.813, 1162.733]	Ref.		<0.0001
	Smoker	490 (21.9%)	748.178 [698.657, 801.209]	-0.584 [-0.696, -0.472]	<0.0001	

AIC	BIC	R2
6785.59	6859.84	0.717

Results are derived from a linear mixed-effect model in which sample's donor was modeled as a random effect to account for intra-individual variability. Anti-spike protein antibody titer was log2-transformed in order ot fit the assumptions of the model. Column *Adj.Means / Partial R2 [95%CI]* displays group means for categorical variables after transforming back the antibody titer to their original scale (Adj.Means), while partial coefficient determinations are reported for continuous variables (Part.R2). **Infected/Nprot+:** previously infected subjects with conserved response against nucleocapsid protein; **Infected/Nprot-:** previously infected subjects with lost response against nucleocapsid protein; **NonVacc:** Non Vaccinated subjects;. **95%CI:** 95% confidence interval; **IQR:** Interquartilic Range; **Adj.Means:** means adjusted by the rest of covariates; **Part.R2:** partial coefficient of determination adjusted by covariates; **Coef:** regression coefficients; **LRT:** Likelihood Ratio Test; **AIC:** Akaike's Information Criterion; **BIC:** Bayes Information Criterion; **R2:** coefficient of determination for the full model.

Table S3. Fold-changes (FC), 95% Confidence Intervals (95%CI) and p-values for association of antibody response against spike protein with clinical variables previously linked to poor outcome in SARS-CoV-2 infection.

	Sex Female		Age (per 5 years)		Body Mass Index (per 5 points)		Arterial Hypertension		Smoking Habit	
	FC [95%CI]	P-value	FC [95%CI]	P-value	FC [95%CI]	P-value	FC [95%CI]	P-value	FC [95%CI]	P-value
**Infected Nprot+ NotVacc**	1.11 [-1.2, 1.46]	0.4776	1.05 [-1.00, 1.10]	0.0601	1.52 [1.31, 1.77]	< 0.0001	1.24 [-1.47, 2.27]	0.4771	-1.65 [-2.36, -1.15]	0.0067
**Infected Nprot+ BNT162b2**	1.01 [-1.20, 1.23]	0.8964	1.06 [1.03, 1.10]	0.0005	1.20 [1.10, 1.32]	< 0.0001	1.67 [1.24, 2.26]	0.0008	-1.59 [-2.04, -1.24]	0.0003
**Infected Nprot+ mRNA-1273**	1.31 [-1.10, 1.90]	0.1486	1.07 [1.01, 1.14]	0.0329	1.21 [1.05, 1.40]	0.0100	1.30 [-1.29, 2.16]	0.3193	-1.62 [-2.40, -1.10]	0.0160
**Infected Nprot- BNT162b2**	1.86 [-1.07, 3.70]	0.0758	1.03 [-1.09, 1.17]	0.5878	1.02 [-1.49, 1.56]	0.9150	-1.58 [-7.15, 2.88]	0.5549	-1.73 [-3.18, 1.06]	0.0752
**Non-Infected BNT162b2**	-1.18 [-1.31, -1.06]	0.0021	-1.09 [-1.11, -1.07]	< 0.0001	-1.00 [-1.05, 1.05]	0.9696	-1.11 [-1.31, 1.06]	0.1895	-1.59 [-1.75, -1.45]	< 0.0001
**Non-Infected mRNA-1273**	1.19 [-1.06, 1.50]	0.1470	-1.03 [-1.07, 1.01]	0.1106	1.06 [-1.06, 1.18]	0.3284	1.08 [-1.21, 1.41]	0.5609	-1.09 [-1.31, 1.11]	0.3900

For each variable, estimations are derived from a linear mixed-effect model in which sample's donor was modeled as a random effect to account for intra-individual variability, and are adjusted by the rest of confounder variables (sex, age, body mass index, arterial hypertension, smoker habit and time intervals from serology and infection and vaccination). In each case, the model included the interaction with the variable bein avaluated in order to conduct comparisons within each of the subject groups. Anti-spike protein antibody titer was log2-transformed in order ot fit the assumptions of the model. To facilitate the interpretation of the results, FCs lower than 1 have been reversed (1/FC) and flagged with a minus sign ("-"). **Infected/Nprot+:** previously infected subjects with conserved response against nucleocapsid protein; **Infected/Nprot-:** previously infected subjects with lost response against nucleocapsid protein; **NonVacc:** Non Vaccinated subjects.

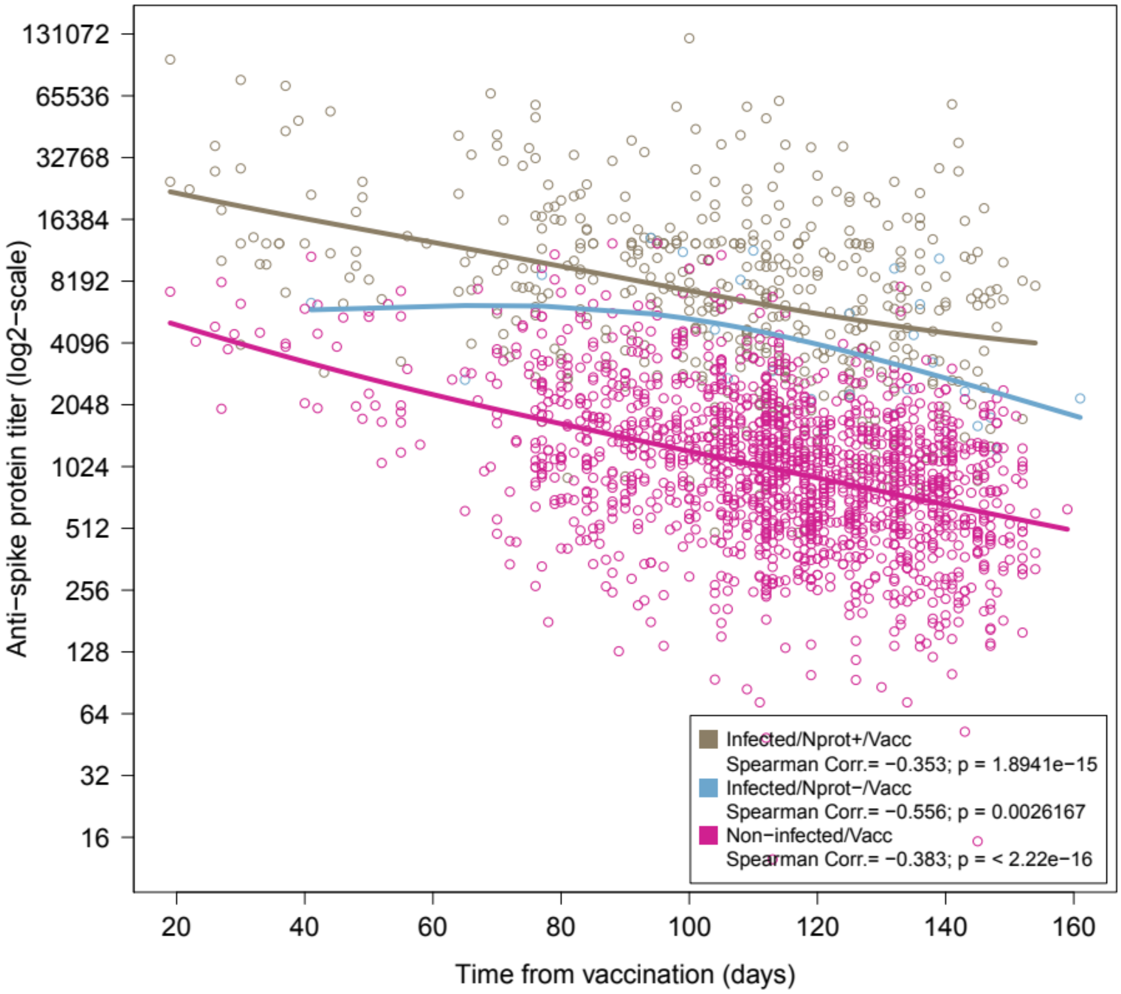

Figure S1: Scatter plot showing the relationship of anti-spike antibody titers (log2-scale) with time from vaccination to serology (days) in SARS-CoV-2 vaccinated subjects. **Vacc:** subjects vaccinated with mRNA SARS-CoV-2 vaccine; **Spearman.Corr.:** Spearman correlation coefficient; **p**: p-value of Spearman Correlation.

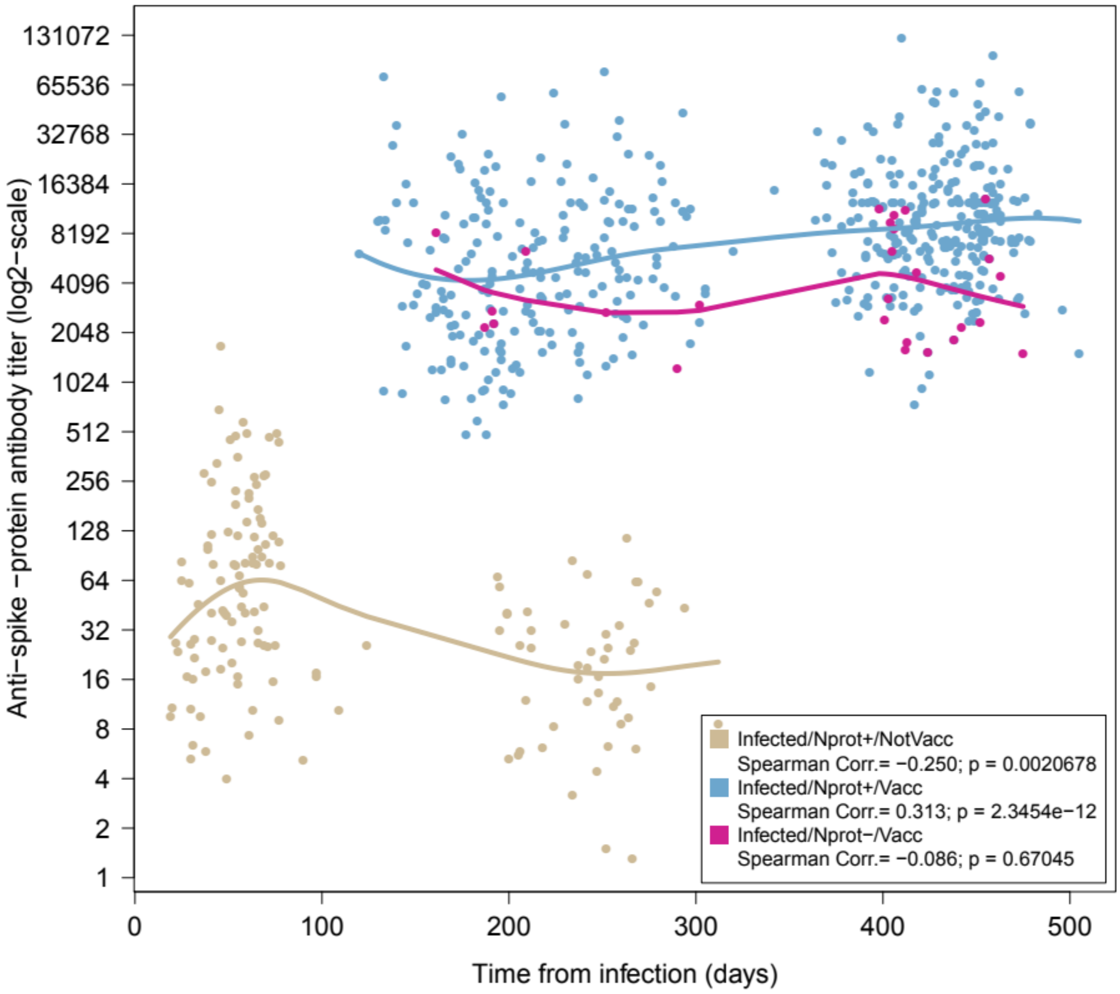

Figure S2: Scatter plot showing the relationship of anti-spike antibody titers (log2-scale) with time from infection to serology (days) in previously SARS-CoV-2 infected subjects. **Vacc:** subjects vaccinated with mRNA SARS-CoV-2 vaccine; **NotVacc:** subjects not vaccinated; **Spearman.Corr.:** Spearman correlation coefficient; **p**: p-value of Spearman Correlation.

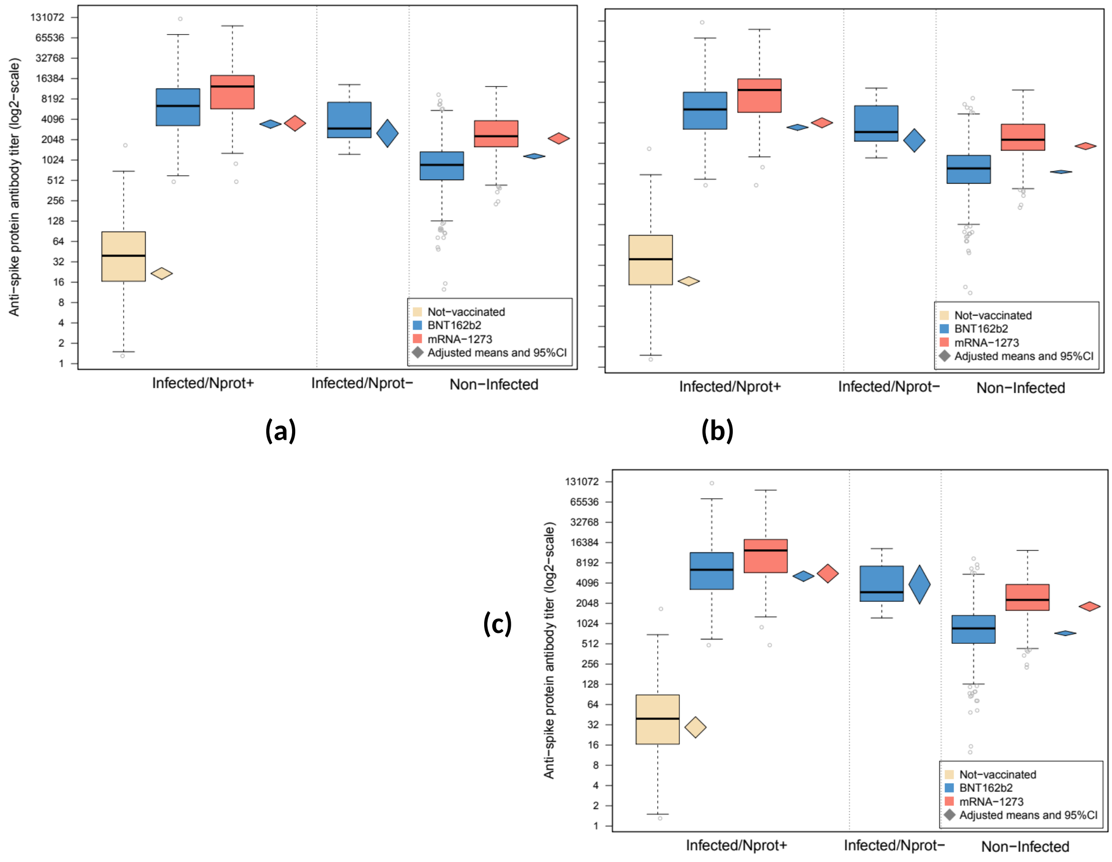

Figure S3. Antibody response against SARS-CoV-2 spike protein in cohort subjects aged from **(a)**18 to 34, **(b)** 35 to 54 and **(c)** 55 to 70. Subjects were grouped according to their infection and conservation status of antibody response against nucleocapsid protein. Boxplots represent the distribution of anti-spike protein antibody quantification. Upper and lower bounds of boxes indicate percentiles 75 and 25, respectively. Whiskers extends 1.5 times the interquartilic range (IQR) from each extreme of the boxes. Diamond-shape symbols represent the adjusted group means of anti-spike protein antibody titer after statistical control for confounders, and their extension represent their 95% confidence intervals. These estimations were derived from a linear mixed-effect model in which sample's donor was modeled as a random effect to account for intra-individual variability, and were adjusted by sex, body mass index, arterial hypertension, smoking habit and time interval from infection and/or vaccination to serology. Anti-spike protein antibody quantifications are expressed in log2-scale. **95%CI:** 95% confidence interval.
